# Do you trust me? Driver responses to automated evasive maneuvers

**DOI:** 10.3389/fpsyg.2023.1128590

**Published:** 2023-05-31

**Authors:** Nicholas Britten, Mishel Johns, Jon Hankey, Ko Kurokawa

**Affiliations:** ^1^Virginia Tech Transportation Institute, Blacksburg, VA, United States; ^2^Virginia Polytechnic Institute and State University, Department of Industrial and Systems Engineering, Blacksburg, VA, United States; ^3^Ford Motor Company, Palo Alto, CA, United States; ^4^Ford Motor Company, Dearborn, MI, United States

**Keywords:** conditional automation, driver intervention, evasive maneuver, driver eye glance, driver trust

## Abstract

An increasing number of Conditionally Automated Driving (CAD) systems are being developed by major automotive manufacturers. In a CAD system, the automated system is in control of the vehicle within its operational design domain. Therefore, in CAD the vehicle is capable of tactical control of the vehicle and needs to be able to maneuver evasively by braking or steering to avoid objects. During these evasive maneuvers, the driver may attempt to take back control of the vehicle by intervening. A driver interrupting a CAD vehicle while properly performing an evasive maneuver presents a potential safety risk. To investigate this issue, 36 participants were recruited to participate in a Wizard-of-Oz research study. The participants experienced one of two evasive maneuvers of moderate intensity on a test track. The evasive maneuver required the CAD system to brake or steer to avoid the box placed in the lane of travel of the test vehicle. Drivers glanced toward the obstacle but did not intervene or prepare to intervene in response to the evasive maneuver. Importantly, the drivers who chose to intervene did so safely. These findings suggest that after experiencing a CAD vehicle for a brief period, most participants trusted the system enough to not intervene during a system-initiated evasive maneuver.

## Introduction

Many of today’s vehicles offer Society of Automotive Engineers (SAE) Level 2 or Partially Automated Driving (PAD) features while an increasing number of SAE Level 3 or Conditionally Automated Driving (CAD) are being developed by major automotive manufacturers (e.g., Mercedes-Benz; Perez, 2022). SAE J3016 provides a taxonomy of the levels of driving automation and the role of the human driver and the driving automation system within each level (SAE, 2021). PAD is defined as a driver support feature wherein the driving automation system supports the driver by providing longitudinal and lateral control inputs. While the driver is expected to monitor the roadway environment and is required to supervise the driving automation system. During PAD the driver must be ready to resume manual control of the vehicle at all times. In contrast to PAD, during CAD, the automated driving system is capable of performing the entire driving task within its operational design domain (ODD). This means that the driver is not expected to monitor the roadway environment and is not required to supervise the driving automation system while CAD is engaged. However, the driver is expected to manually control the vehicle or resume monitoring in response to system-initiated requests to take over.

The changes in the role and responsibilities of the driver and driving automation system when shifting from manual driving to PAD (i.e., supervised automation) and CAD (i.e., unsupervised automation) is associated with reduced driver situational awareness or entering an “out-of-the-loop” state (Endsley and Kiris, 1995). The out-of-the-loop state is defined by Merat et al. (2019) as, “Not in physical control of the vehicle, and not monitoring the driving situation, OR in physical control of the vehicle but not monitoring the driving situation.” Conversely, being in physical control of the vehicle and monitoring the driving is defined as the “in-the-loop” state. Monitoring the environment but not being in physical control of the vehicle is defined as the “on-the-loop” state. Due to the expectation for the driver to resume manual control while in CAD and shift to an “in-the-loop” state from an “on-the-loop” or “out-of-the-loop” state, a significant portion of the human factors research focused on the higher levels of driving automation systems has focused on the resumption of manual control (Seppelt and Victor, 2016; Louw et al., 2020). In contrast, there has been limited research on how drivers respond to evasive maneuvers initiated by a CAD vehicle.

In CAD, the automated system is in control of the vehicle within its ODD. This means that during CAD the vehicle is capable of tactical control of the vehicle and can maneuver evasively by braking or steering to avoid objects on the road. During these evasive maneuvers, the driver may attempt to intervene during the maneuver and take back control of the vehicle. The interruption of a properly performed evasive maneuver may reduce safety with a CAD system. Additionally, unexpected inertial forces might also affect driver inputs on the controls. The potential safety risk of driver intervention during system-initiated critical events (Roche et al., 2020, 2022) and minimal risk maneuvers (Karakaya and Bengler, 2021) have been demonstrated in driving simulators. Roche et al. (2022) found that drivers tended to unsafely depart the travel lane or collide with an obstacle when intervening during a CAD system-initiated critical lane change. While Roche et al. (2020) found that drivers tended to overreact by either decelerating too strongly or unnecessarily changing lanes when intervening during a CAD system-initiated critical braking maneuver. However, both of these studies required the drivers to intervene and take control of the vehicle and did not examine whether drivers will voluntarily choose to intervene during system-initiated maneuvers. Results from studies examining whether drivers would intervene to avoid an object during PAD have generally found that a subset of drivers will not intervene and will subsequently strike the object despite having their eyes on the road and their hands on the wheel (Victor et al., 2018; Pipkorn et al., 2021). Becker et al. (2022) found that drivers choose to intervene during CAD-initiated critical braking maneuvers in response to a cut-in vehicle between 15.7 and 25.5% of the time and that these interventions can lead to collisions. Karakaya and Bengler (2021) found that a high proportion of drivers (i.e., over 50%) chose to intervene during steering and braking minimal risk maneuvers performed by an automated driving system. However, these studies were conducted in driver simulators, which do not always replicate real-world driving behavior. For example, Eriksson et al. (2017) found that drivers exhibited faster reaction times in response to takeover requests in real-world traffic compared to in a driving simulator. Further, Becker et al. (2022) focused on driver interventions to critical maneuvers rather than evasive maneuvers and did not examine whether drivers would choose to intervene in steering maneuvers. While Karakaya and Bengler (2021) examined minimal risk maneuvers, which are meant to bring the vehicle to a safe state and transition to manual driving or a stop if the driver does not intervene, as opposed to evasive maneuvers which are meant to avoid an unsafe situation and continue in automated mode. To the authors’ knowledge, no currently published research indicates how drivers may respond to evasive braking and steering maneuvers initiated by a CAD vehicle.

Presently, CAD vehicles have only limited commercial availability and are not accessible to researchers. Therefore, to study CAD vehicles, researchers often need to simulate driving automation systems using Wizard-of-Oz (WoZ) platforms (Wang et al., 2017; Bengler et al., 2019). The WoZ methodology utilizes a “wizard” (i.e., a hidden human experimenter) to simulate a computer system’s role (Fraser and Gilbert, 1991). In the vehicle research field, the WoZ method has been adopted to simulate driving automation systems that are “not yet existent, or whose implementation would be too costly for the purpose of the experiment” (Jarosch et al., 2019b). This application of the WoZ method is used both in driving simulators (Schieben et al., 2009) and with real vehicles on test tracks (Pipkorn et al., 2021) and on public roads (Jarosch et al., 2019a). Researchers have built numerous other WoZ vehicles to simulate Level 2 through 5 driving automation systems to study a broad array of research topics, including non-driving task engagement during unsupervised automation (Klingegård et al., 2020), drive takeover performance during CAD (Purucker et al., 2018), interactions between vulnerable road users and autonomous vehicles (Rothenbücher et al., 2015; Li et al., 2020), and communication between autonomous vehicles and vulnerable road users via external human-machine interfaces (Chen et al., 2020; Faas and Baumann, 2020).

Understanding how drivers respond to evasive maneuvers initiated by a CAD vehicle is important to the implementation of these systems and the arbitration of controls between the driver and automation in such situations. To explore this issue, 36 participants (18 males, 18 females) were exposed to a system capable of PAD and CAD using a WoZ vehicle to emulate PAD and CAD. The participants experienced one of two evasive maneuvers (braking or steering at about 0.3 g) on a controlled test track. The purpose of the study was to investigate the following research question:

*RQ1*: How will drivers respond to an evasive maneuver scenario when in CAD?

- When the event is a swerve initiated by the system?
- When the event is a braking maneuver initiated by the system?

## Materials and methods

### Participants

#### Participant recruitment

Virginia Tech Institutional Review Board approval was obtained for human participants’ data collection. News and social media advertisements, including posts to the Virginia Tech Transportation Institute’s (VTTI) Facebook page, and email were used to recruit participants. In addition, potential participants were identified in VTTI’s recruitment database, which is a large database of individuals who have previously participated or expressed interest in participating in VTTI research. Potential participants were provided with information about the study over the phone from a member of the VTTI recruitment team. After receiving this information, those who were interested in participating in the study were screened for eligibility. A recruitment team member obtained verbal consent from the participant before administering the eligibility screening.

#### Participant demographics

A total of 36 participants (18 females, 18 males) between the ages of 30–75 years old were recruited from the New River and Roanoke Valley regions of Virginia. Overall, the mean age of the participants was 53.8 years. The mean age for male participants was 54.7 years (*n* = 18) and 52.8 years (*n* = 18) for female participants.

### Materials and equipment

**Figure 1:**
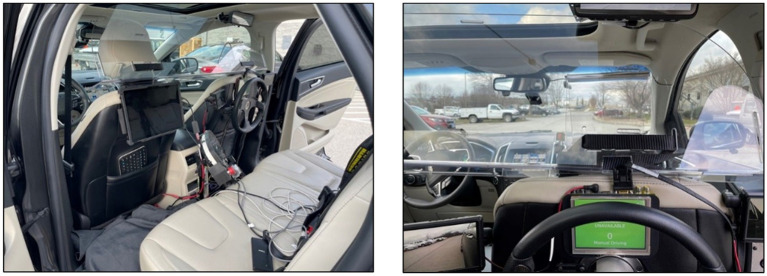
Experimenter workstation and rear-seat driver controls (left), rear-seat driver’s viewpoint while controlling the vehicle (right).

A 2019 Ford Edge was modified to serve as the WoZ test vehicle for this study. The vehicle was equipped with a set of driving controls, displays, to monitor the surrounding environment, and sensors in the rear passenger seat that allowed an experimenter to act as a rear-seat driver (i.e., the “wizard”) and operate the vehicle. The set of controls included a steering wheel, brake and accelerator pedals, turn signals, and buttons to activate the vehicle’s Adaptive Cruise Control (ACC) and Lane Keep Assist (LKAS) features (see Figure 1). These modifications allowed the vehicle to be fully controlled from the rear seat and thereby simulate a vehicle capable of CAD. This simulation was achieved when the wizard steered the vehicle and monitored the driving environment while ACC was active. Although the vehicle was capable of PAD through the simultaneous activation of the ACC and LKAS features, PAD mode was simulated in the same manner as CAD. The vehicle was also equipped with a rear-seat experimenter workstation that allowed a second rear-seat experimenter to control the instrument cluster HMI via a laptop and indicate to the participant when the vehicle was changing between automation modes.

The vehicle was instrumented with VTTI’s FlexDAS data acquisition system (DAS). The DAS had cameras that continuously recorded video of the driver’s face, the forward and rear roadways, an over-the-shoulder view of the driver’s hands and lap area, and the driver’s foot placement from key on to key off. The DAS also recorded vehicle speed, throttle position (front-seat control), brake application (front-seat control), acceleration, turn signal activation, GPS position, steering torque (front-seat control), and automation mode state (i.e., manual driving, PAD, CAD).

### Evasive maneuver

**Figure 2:**
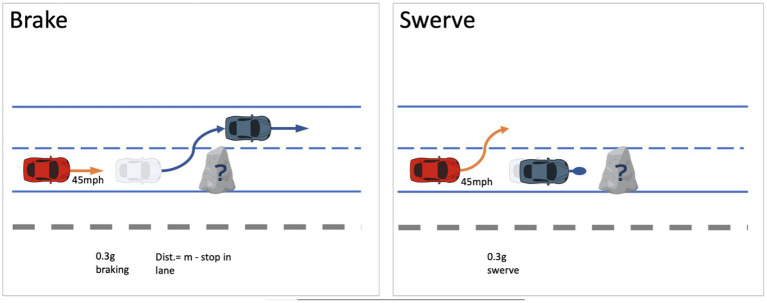
Brake and swerve evasive maneuvers.

The evasive maneuver was performed at the end of the study in a controlled test environment on the highway section of the Virginia Smart Roads. On the second of two laps, the lead vehicle pulled in front of the test vehicle, which was still operating in CAD. Both vehicles were moving at about 45 mph (20 m/s) prior to the maneuver with a headway of 2 s between the vehicles. Once the vehicles reached a set of pre-determined landmarks, which for the test vehicle was approximately 78 m from the box, the rear seat experimenter and lead vehicle initiated opposite evasive maneuvers (e.g., test vehicle brakes and lead vehicle swerves or test vehicle swerves and lead vehicle brakes) in response to a cardboard box in the road (see Figure 2). The vehicles initiated opposite maneuvers to allow the obstacle to be revealed to the test vehicle and for safety reasons (i.e., to reduce the risk of forward collision between the test and lead vehicle). The participant did not need to intervene in any way to avoid the box. The brake maneuvers were designed for the rear experimenter to bring the vehicle to a complete stop ~9 m in front of the box. The swerve maneuvers were designed for the rear experimenter to drive the vehicle around the box and, after passing the box, safely stop the vehicle in the left lane. These maneuvers were intended to mimic an evasive action, where the CAD system detects an object in the road and brakes/swerves to avoid it, as opposed to a “panic” or “emergency” action. Therefore, the velocity, distance to the obstacle, and the lateral and longitudinal acceleration forces used by the test vehicle were scaled to match this evasive nature of the maneuvers, with braking maneuvers of about 0.3 g and lateral swerves of about 0.3 g.

### Procedure

Participation in the study consisted of a single approximately 3-h session during daylight hours that was comprised of four principal stages: (1) pre-drive consent and training, (2) vehicle orientation, (3) on-road driving, and (4) test track driving.

#### Pre-drive consent and training

When the participants arrived at VTTI written consent was obtained from the participant. The participant was given the opportunity to review the consent form and a VTTI researcher answered all the participant’s questions. Upon obtaining consent, the participant’s hearing via an informal “call-and-response” test, visual acuity via a Snellen eye chart, and color vision via an Ishihara test were assessed. The participants were required to pass the hearing test (pass/fail) and the visual acuity test (≥20/40), if participants did not pass these tests, they were informed they did not meet the study requirements and were compensated $30.

Following the consent process, the researcher explained the vehicle’s automated driving features and how to activate and use them safely while driving, including normal operation and possible system limitations. Specifically, participants were instructed that during PAD they must keep their eyes and mind on the driving environment but that they did not need to keep their hands on the steering wheel or feet on the pedals. In addition, during CAD, participants were instructed that they did not need to keep their eyes and mind on the road, their hands on the wheel, or their feet on the pedals.

#### Vehicle orientation

Next, the researcher oriented the participant to the driver’s controls in the vehicle (e.g., seat, steering wheel, and mirror adjustment controls). While reviewing the vehicle controls, the researcher informed the participant about the presence of the second experimenter and their role as the rear driver. Specifically, the researcher explained that the vehicle was equipped with a second set of controls that could be used to take control of the vehicle if necessary (e.g., in a safety situation) and to augment the automation if needed but that the participant was considered the primary driver, who would be responsible for controlling the vehicle, following all roadway signs, and responding to system requests as needed throughout the study. Additionally, participants were instructed that breaks would be provided to them if needed.

#### On-road driving

**Figure 3:**
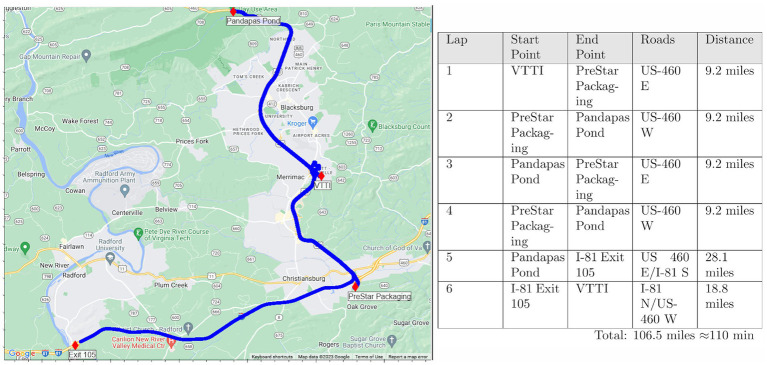
On-road study route overview.

Once the participant felt comfortable with the vehicle’s controls, they were allowed to practice driving the vehicle in the parking lot at VTTI. When the participant indicated they were comfortable with driving the vehicle then they were directed to the public roadway where they experienced 20 transitions between CAD, PAD, and manual driving modes. CAD was only available within the restricted access highway sections of the route. PAD was available during sections of unrestricted access highway but required the vehicle to be traveling at least 45 mph before it could be activated. Manual driving was required when the vehicle exited the highway sections of the route (i.e., when the vehicle turned around at PreStar Packaging, Pandapas Pond, or I-18 Exit 105). Transitions between the driving modes occurred when there were transitions between these road types (e.g., road change from restricted access to unrestricted access highway resulted in a transition between CAD to PAD). The route was designed so that the vehicle was in CAD during the majority of the route. Participants drove 6 laps on public roads over an approximately 2-h time period (see Figure 3).

#### Test track driving

**Figure 4:**
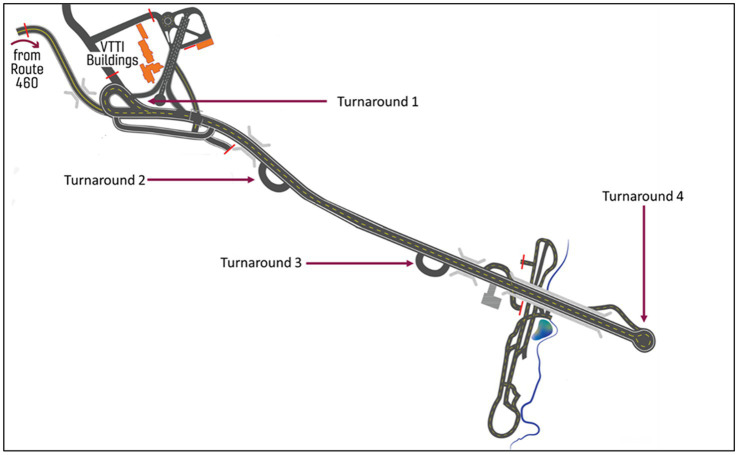
Map of the highway portion of the Virginia Smart Roads test track.

Upon completing the final lap on the public road, the study continued with a test track portion on the highway section of the Virginia Smart Roads. After entering the test track, participants were instructed that the study would continue with a few laps on the test track to make sure the participant could experience all the maneuvers the system was capable of and that another vehicle (i.e., the lead vehicle) would also be on the road. The test track portion of the study consisted of two laps, proceeding from the 1st turnaround to the 4th turnaround (see Figure 4). During the 1st lap, the CAD system was activated once the vehicle reached a speed of 45 mph. As the vehicles continued driving the lead vehicle activated its turn signal and pulled to the side of the roadway near the 3rd turnaround while the test vehicle continued to drive past it, turn around at the 4th turnaround, and proceed back up the road. Once the test vehicle passed out of sight of the lead vehicle, the experimenter in the lead vehicle placed the obstacle in the roadway, drove to the 2nd turnaround, and waited for the test vehicle to turn around at the 1st turnaround and head in their direction. When the test vehicle arrived back at the 2nd turnaround the lead vehicle pulled out and drove ahead of it. Once the test vehicle reached a pre-determined landmark approximately 78 m from the box, the rear seat experimenter and lead vehicle initiated opposite evasive maneuvers (e.g., test vehicle brakes and lead vehicle swerves or test vehicle swerves and lead vehicle brakes) in response to a cardboard box in the road. For the evasive steering maneuver, the rear seat experimenter brought the vehicle to a stop in the lane after passing by the obstacle whereas the rear seat experimenter brought the vehicle to a stop in front of the box for the evasive braking maneuver. After the evasive maneuver, the rear-seat experimenter brought the vehicle to a complete stop, explained to the participants that the maneuver was a planned portion of the study, and exited the Smart Roads test track, completing the test track portion of the study. After completing the test-track portion of the study, the participant was thanked for their time and provided with compensation via a MasterCard pre-loaded with $100.

## Results

### Data annotation and analysis

Vehicle data from 36 participants were collected and analyzed. There is timestamped data for each participant that includes:

- Acceleration, speed, orientation, lat/long coordinates
- Rear driver (experimenter) brake torque, steering torque, steering angle, throttle percentage
- Front driver (participant) brake torque, steering torque, steering angle, throttle percentage
- Intervention flag – when the automated system was canceled with a button or brake

**Figure 5:**
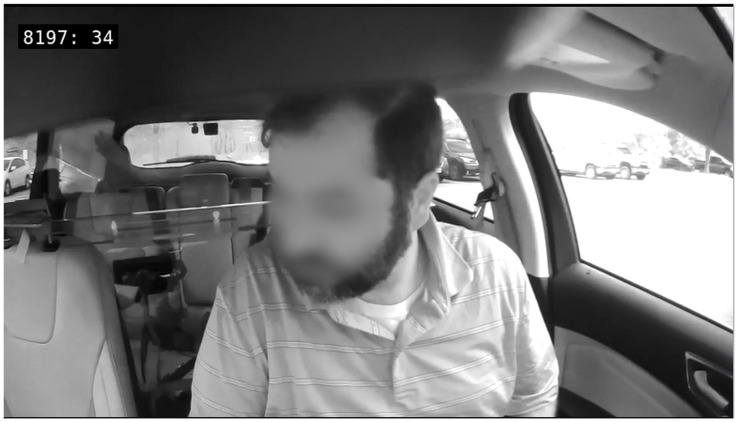
Example of DAS face camera.

In addition, behavioral data from 34 participants were obtained via annotation of the video data. Two of the participants were excluded from the analysis because a DAS error made their video data unavailable. The annotation was performed by a trained VTTI data reductionist, and eye glance data was captured using the DAS face camera (see Figure 5 below). The location of each glance was measured frame-by-frame by trained VTTI data reductionists using the origin method (ISO, 2020). In addition, the participants’ steering wheel and brake pedal behavior (i.e., whether the participant reached toward the steering wheel/brake pedal or had their hands on the wheel/feet on the brake) was measured by annotating the over-the-shoulder and foot camera view videos. Brake pedal behavior could not be measured for three participants because the foot camera view was misaligned during their sessions.

Data were collected from the event start to the event end. Event start was defined as when the test vehicle reached a pre-determined landmark ~106 m before the object placed in the roadway. This distance was selected to capture the participants’ behavior in the seconds leading up to the start of the maneuver. Event end was defined as when the vehicle velocity reached 0 mph for the braking maneuvers or when the front of the vehicle was even with the object, as determined by the front video view for the swerving maneuvers. The start of the experimenter-initiated braking or steering was identified through the Rear Driver brake torque and steering torque variables.

The evasive maneuver data was analyzed in the R and Python statistical computing and graphics environments. JMP Pro 16 was used to perform the chi-square tests of independence.

### Interventions

A chi-square test of independence showed that there was no significant association between maneuver type and the number of participants who chose to intervene during the evasive maneuver, χ^2^ (1, N = 34) = 0.007, *p* = 0.93. Only two participants chose to intervene and deactivate the system during the evasive maneuver – one participant each in the swerve and brake conditions. Both participants used the brake pedal to deactivate the system and moved their hands to the steering wheel when they deactivated the system. Both participants had their hands off the wheel prior to the event.

### Reaching for controls

#### Steering wheel

**Figure 6:**
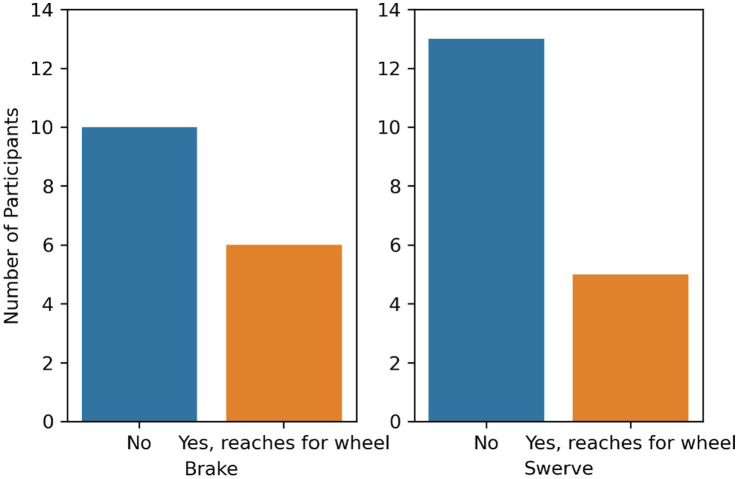
Number of participants that reached for the steering wheel by maneuver type.

A chi-square test of independence showed that there was no significant association between maneuver type and the number of participants reaching toward the steering wheel, χ^2^(1, *N* = 34) = 0.37, *p* = 0.55. 37% of the participants in the brake condition (*n* = 16) and 27% of the participants in the swerve condition (*n* = 18) reached for the steering wheel during the maneuver (see Figure 6).

#### Brake

**Figure 7:**
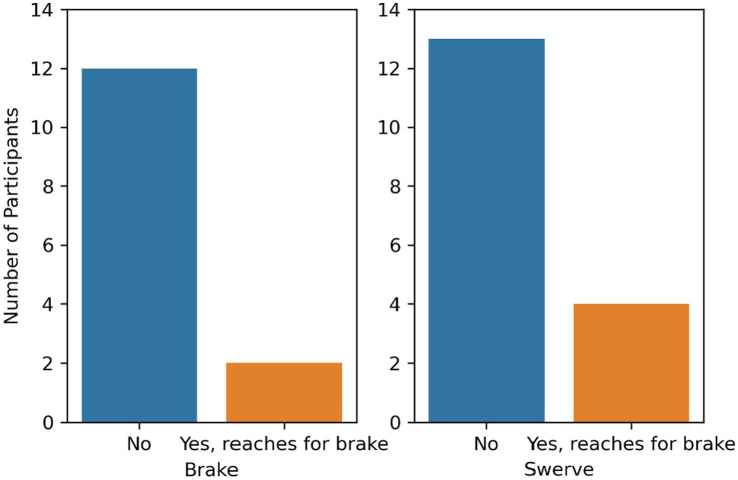
Number of participants that reached for the brake pedal by maneuver type.

A chi-square test of independence showed that there was no significant association between maneuver type and the number of participants reaching toward the brake pedal, χ^2^(1, *N* = 34) = 0.43, *p* = 0.51. Specifically, 14% of the participants in the brake condition (*n* = 16) and 22% of the participants in the swerve condition (*n* = 18) reached for the brake pedal (see Figure 7).

#### Cancel button

No participants in either the brake or swerve condition used the cancel button or reached for the cancel button.

### Intervention capability

**Figure 8:**
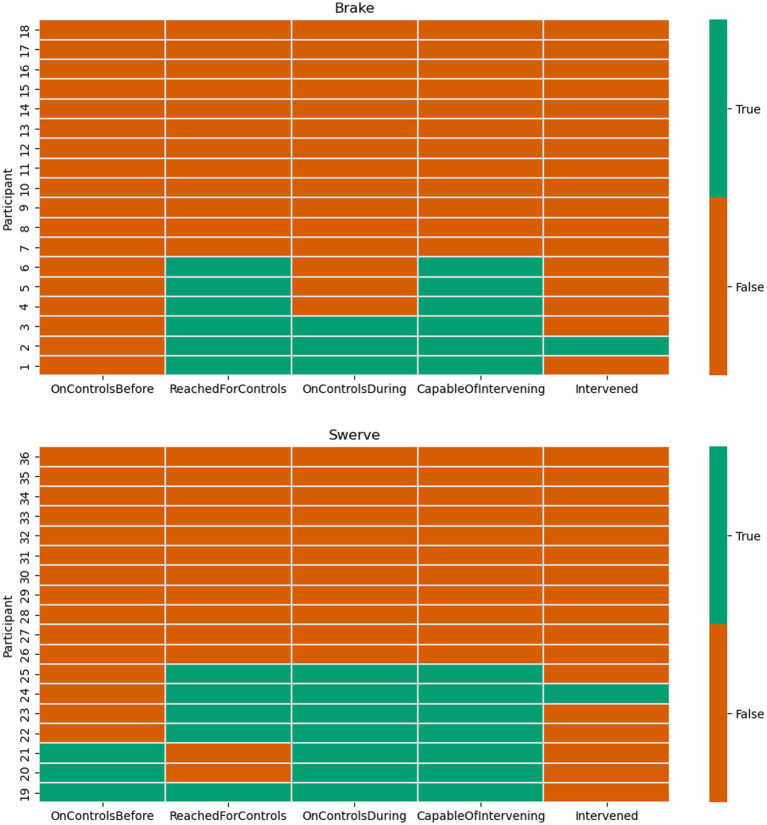
Participants’ intervention capability during the maneuver.

The number of participants who were capable of intervening was defined as the number of participants who had their hands on the wheel or feet on the brake before the maneuver or who reached to the wheel or brake during the maneuver. In total, 13 participants were classified as ready to intervene with six participants in the braking condition and seven in the swerve condition (see Figure 8).

### Eye glance

**Figure 9:**
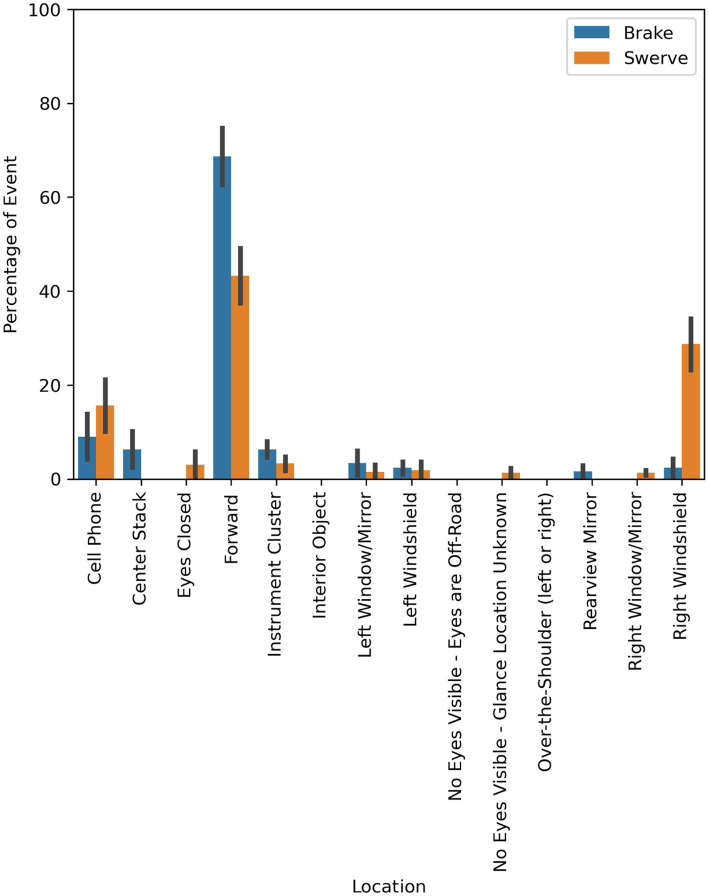
Mean proportion of glance duration to areas of interest by maneuver type.

As illustrated in Figure 9, participants in both evasive maneuver conditions primarily looked toward the obstacle and the lead vehicle. This is reflected in the proportion of total time spent looking forward in the brake condition (68.69%; SD = 34.19) and the total time spent looking forward (43.29%; SD = 34.91) and at the right windshield (28.71%; SD = 32.67) in the swerving condition. The mean duration of the braking maneuvers was ~9.5 s (SD = 1.6) and the mean duration of the swerve maneuvers was ~6.2 s (SD = 0.4).

**Figure 10:**
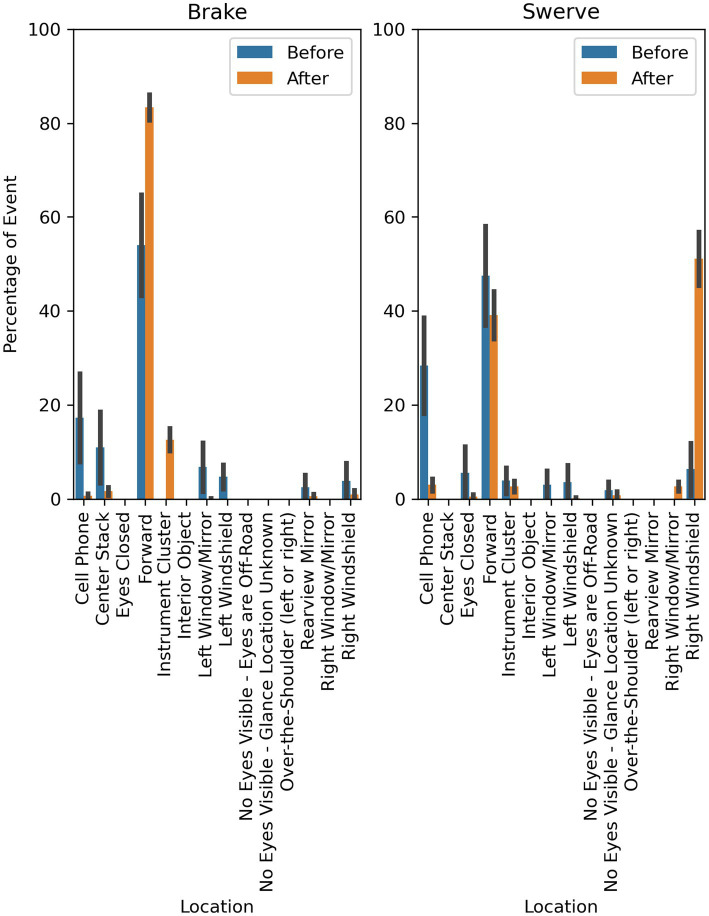
Mean proportion of eye glance duration before and after maneuver by maneuver type.

This pattern is further illustrated in Figure 10, which shows the mean proportion of time spent glancing to the areas of interest before and after the maneuver occurred for each maneuver type. Participants in the brake condition had a mean increase of 29.4% of time spent looking at the forward roadway after the maneuver (83.38%; SD = 10.97) compared to before (53.98%; SD = 42.84). The mean duration of the event before the braking maneuver was 1.8 s (SD = 0.4) and 7.7 s (SD = 1.7) after. Participants in the swerve condition had a mean increase of 44.75% of time spent looking to the right windshield after the maneuver (51.08%; SD = 24.17) compared to before (6.33%; SD = 24.27). The mean duration of the event before the swerve maneuver was 1.2 s (SD = 0.4) and 5.0 s (SD = 0.4) after.

On an individual level, 65% of participants (22/34) were looking toward the forward-driving environment (i.e., glancing at the forward, left windshield, or right windshield locations) immediately before the beginning of the evasive maneuver with 11 participants in the brake condition and 11 participants in the swerve condition. Conversely, 35% of participants (12/34) were looking away from the forward-driving environment (i.e., not glancing at the forward, left windshield, or right windshield locations) immediately before the beginning of the evasive maneuver with five participants in the brake condition and seven participants in the swerve condition. 36% of participants (8/22) who were looking forward before the maneuver reached for the wheel (five brake, three swerve), and two of these participants intervened. While 25% of participants (3/12) who were looking away from the forward driving environment reached for the wheel (one braking, two swerving), and zero of these participants intervened.

### Acceleration data

#### Lateral acceleration

**Figure 11:**
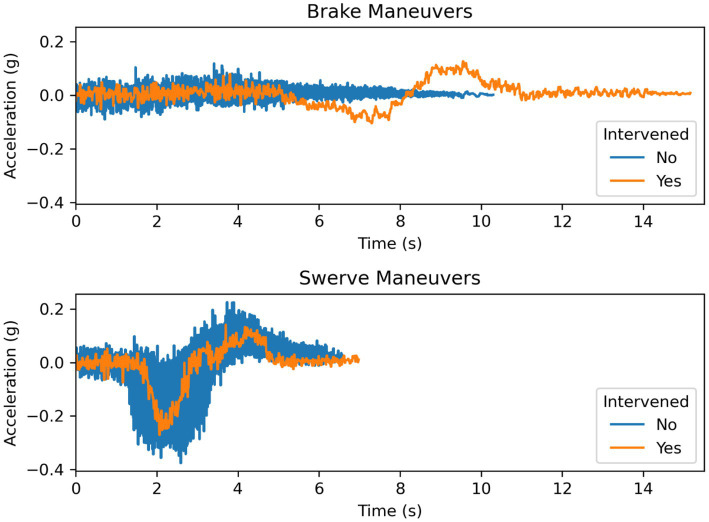
Lateral acceleration during braking and swerving maneuvers.

The mean maximum lateral acceleration for the swerve maneuvers was 0.148 g (SD = 0.03) when the participants did not intervene and 0.141 g (SD = NA) when the participant intervened. The mean maximum lateral acceleration for the brake maneuvers was 0.08 g (SD = 0.031) when the participants did not intervene and 0.126 g (SD = NA) when the participant intervened. The mean minimum lateral acceleration for the swerve maneuvers was −0.293 g (SD = 0.049) when the participants did not intervene and −0.269 g (SD = NA) when the participant intervened. The mean minimum lateral acceleration for the brake maneuvers was −0.066 g (SD = 0.009) when the participants did not intervene and −0.104 g (SD = NA) when the participant intervened (Figure 11).

#### Longitudinal acceleration

**Figure 12:**
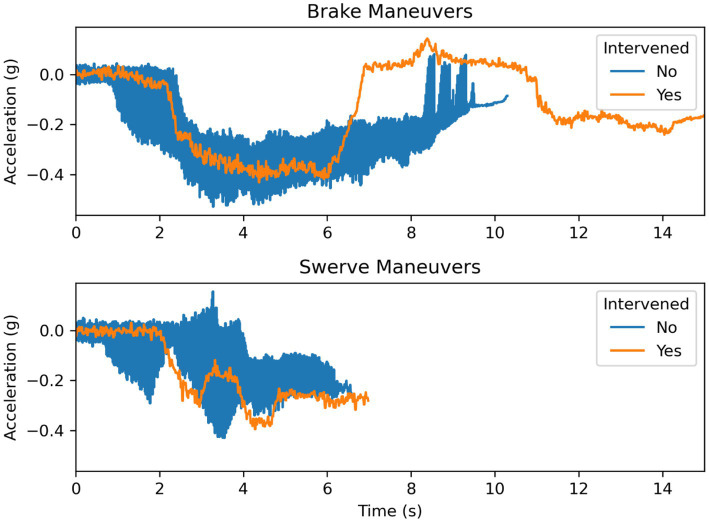
Longitudinal acceleration during braking and swerving maneuvers.

The mean maximum longitudinal acceleration for the swerve maneuvers was 0.047 g (SD = 0.03) when the participants did not intervene and 0.030 g (SD = NA) when the participant intervened. The mean maximum longitudinal acceleration for the brake maneuvers was 0.04 g (SD = 0.019) when the participants did not intervene and 0.144 g (SD = NA) when the participant intervened. The mean minimum longitudinal acceleration for the swerve maneuvers was −0.27 g (SD = 0.055) when the participants did not intervene and −0.39 g (SD = NA) when the participant intervened. The mean minimum longitudinal acceleration for the brake maneuvers was −0.44 g (SD = 0.054) when the participants did not intervene and −0.431 g (SD = NA) when the participant intervened (Figure 12).

## Discussion

Overall, the results from this study show that most drivers do not prepare to intervene or intervene in response to a CAD system-initiated evasive maneuver. Indeed, only 11 participants reached toward the vehicle controls (i.e., prepared to intervene) and, of the few participants that did reach for the controls, only two participants chose to intervene. This is a low number of interventions in comparison to previous research on driver responses to critical braking maneuvers (Becker et al., 2022) and braking and steering minimal risk maneuvers (Karakaya and Bengler, 2021). The difference in the number of interventions shows that drivers may be less likely to intervene during CAD-initiated evasive maneuvers than during critical maneuvers. Critical maneuvers occur at higher longitudinal and lateral acceleration with shorter times and distances between vehicles compared to evasive maneuvers. The evasive maneuvers in this study had a mean time headway of 1.5 s between the test vehicle and the box in the road when the maneuver was initiated. By way of comparison, the critical maneuvers in the Becker et al. (2022) study ranged from 0.31 to 0.05 s. Driver preparation to intervene, as measured by reaching toward the steering wheel, brake pedal, or cancel button, in response to these maneuvers is similar regardless of whether the vehicle brakes or swerves to execute the evasive maneuver. Across both maneuver types, more drivers reached for the steering wheel than the brake pedal. This finding could be due to drivers having different trust in the braking and steering capabilities of the CAD system. For example, drivers might have trusted the system’s ability to brake more than the system’s ability to steer, resulting in more drivers reaching for the steering wheel than the brake. This finding could also be because the procedure emphasized that braking disables the CAD system. For example, if the driver brakes, they are asking for control of the vehicle. In contrast, if the driver holds onto the steering wheel, they are preparing to intervene but have not explicitly disengaged the CAD system. In addition, the results show that the type of evasive maneuver does not appear to affect the driver’s choice to intervene and take control of the vehicle during CAD system-initiated maneuvers.

Interestingly, most drivers were monitoring the roadway (i.e., looking toward the forward roadway, left windshield, or right windshield locations) immediately before the beginning of the evasive maneuver. In other words, drivers were looking toward the roadway and elected not to intervene or prepare to intervene. The drivers’ glance behavior also showed that the drivers tended to appropriately attend to the evasive maneuver. Specifically, drivers in the brake condition shifted their visual attention to the forward roadway (i.e., the swerving lead vehicle and box in the lane of travel) in response to maneuver while drivers in the swerve condition shifted their visual attention to the right windshield (i.e., toward the box in the lateral lane).

Beyond these overall results, it is also important to characterize the behavior of the intervening drivers. Both drivers were capable of intervention before deciding to deactivate the system. The intervening drivers reached for the steering wheel 1.7 and 0.46 s after the initiation of the braking and swerving maneuvers, respectively. Additionally, these drivers reached for the brake pedal 2.1 and 0.2 s after the initiation of the braking and swerving maneuvers, respectively. Both drivers chose to disengage the system by pressing the brake pedal 3.0 and 2.31 s after the maneuver was initiated during the braking and swerving maneuvers, respectively. Neither of the participants elected to use the cancel button on the steering wheel to disengage the system. This is likely because the experiment emphasized the brake pedal disengagement method to the participant during the pre-drive training.

**Figure 13:**
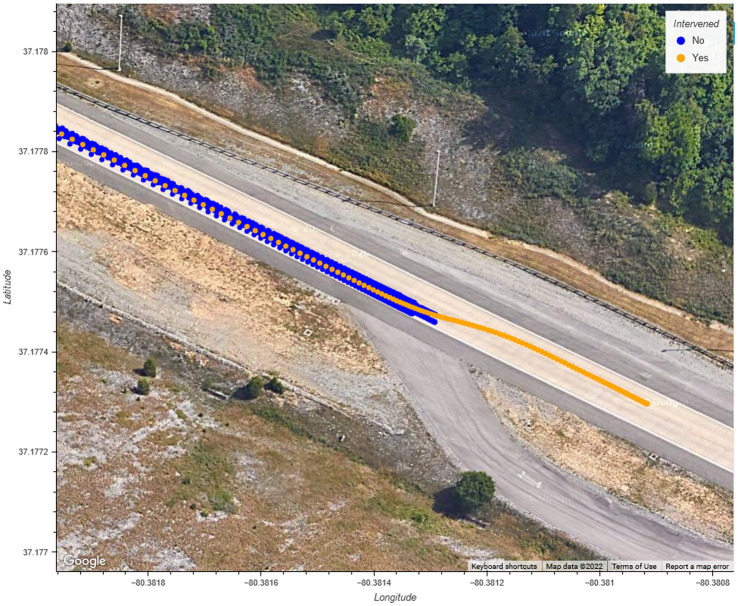
GPS position of the vehicle during braking evasive maneuvers.

**Figure 14:**
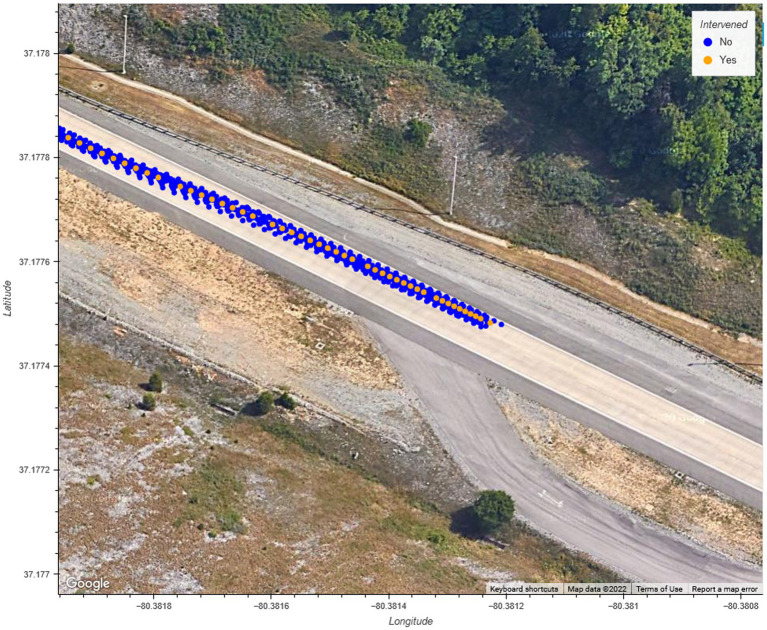
GPS position of the vehicle during swerving evasive maneuvers.

As illustrated in Figure 13, the driver who intervened during the braking maneuver chose to steer around the obstacle by changing into the adjacent lane before bringing the vehicle to a stop. It is possible that the driver was following the behavior of the lead vehicle, which swerved to maneuver around the obstacle. The driver who intervened during the swerving maneuver disengaged the system after the vehicle was maneuvering into the adjacent lane but did not apply steering input until the vehicle finished changing lanes. The vehicle, as shown in Figure 14, followed the same trajectory as the vehicle under system control (i.e., during the non-intervening maneuvers) before safely stopping the vehicle. The results from the acceleration data show that the peak lateral and longitudinal acceleration were largely similar during the driving interventions and the system-controlled events. In combination, these results show that the driver interventions were safe. Additionally, these results indicate drivers do not overact by over-correcting steering or braking too strongly in reaction to sudden system-initiated braking and steering maneuvers.

Previous research has indicated that higher trust in automation is correlated with longer takeover times (Payre et al., 2016; Körber et al., 2018) and driver inaction (Victor et al., 2018) during takeover events. While lower trust in automation is associated with a higher probability of driver-initiated takeovers from CAD during critical maneuvers (Becker et al., 2022). Although trust was not assessed by a questionnaire in this study, these previous findings indicate that the general lack of driver intervention and inaction in response to the evasive maneuvers observed in this study meant that the drivers trusted the system. Taken together these results suggest that after just a couple of hours of driving on public roads, most participants trusted the system enough to not intervene during a maneuver in response to a sudden-reveal road hazard.

## Limitations

One limitation of the WoZ method is that it is difficult for a human driver to re-create the precision of automation when repeatedly positioning a vehicle (Klingegård et al., 2020). Several efforts were made to address this limitation. PAD and CAD were achieved by using the test vehicle’s ACC longitudinal control system in combination with manual steering, thereby removing some human control variability. A single rear-seat experimenter served as the wizard, increasing the consistency of the experience between participants. Despite these efforts, it is possible that the wizard was not able to completely mimic an automated driving system for the entirety of the study. The participants’ perception of the WoZ vehicle was not qualitatively assessed at the end of the study. Previous research has found that some study participants perceive a WoZ vehicle as automated even when the platform is designed not to employ deception (Baltodano et al., 2015). However, with this being said, it is possible that the presence of the rear seat experimenters in this study was a potentially confounding variable.

Due to the low number of interventions, only one driver intervention acceleration profile was available in each of the maneuver conditions. Therefore, these acceleration profiles are example responses not necessarily indicative of the general driver population. Future research should be conducted to assess the safety of driver responses to CAD-initiated evasive maneuvers that includes acceleration profiles from a greater number of intervening drivers.

## Conclusion and future research

The purpose of this study was to understand how drivers respond to evasive maneuvers initiated by a CAD vehicle. To address this research gap, 36 participants (18 males, 18 females) were exposed to a system capable of PAD and CAD using a WoZ vehicle to emulate PAD and CAD. The participants experienced one of two evasive maneuvers (braking or steering at about 0.3 g) on a controlled test track. Results indicated that in a majority of cases, drivers glanced toward the obstacle after the vehicle initiated the evasive maneuver but did not intervene or prepare to intervene. When drivers chose to intervene, they deactivated the CAD system by pressing the brake and reached their hands to the steering wheel. The participants who did intervene were safe while doing so. It is important to note, though, that the maneuver, while sudden, was conducted at about 0.3 g and not close to the limit of vehicle handling. No differences were found between driver responses in the swerve vs. the braking conditions, other than the direction of glances (aimed at the road hazard). These findings suggest that after experiencing a CAD vehicle for only a couple of hours on public roads, most participants trusted the system enough to not intervene during a maneuver involving moderately high accelerations in response to a sudden-reveal road hazard.

Given the ability of CAD equipped vehicles to operate in PAD and manual modes and the findings from Pipkorn et al. (2021) and Victor et al. (2018) suggesting that drivers do not intervene to avoid obstacles when in PAD, future research should consider the impact of experiencing CAD system-initiated maneuvers on participants’ intervention capability and decision making during subsequent obstacle avoidance scenarios when in PAD.

CAD can lead to a monotonous situation inducing driver fatigue and subsequently reducing driver takeover performance (Jarosch et al., 2019a). However, the impact of driver fatigue on driver responses to CAD-initiated evasive maneuvers is unknown. Future research should examine the relationship between fatigue and driver behavior during CAD-initiated evasive maneuvers.

The current study examined driver responses to a CAD-initiated maneuver on a closed test track with a single additional vehicle. Previous research has shown that the complexity of a traffic situation impacts driver takeover performance (Gold et al., 2016). It follows that driver responses to evasive maneuvers in more complex scenarios may differ from those observed in this study. Future research should examine how drivers respond to evasive maneuvers in more complex traffic situations. In addition, the test and lead vehicles performed opposite evasive maneuvers. It is possible that driver behavior could differ if the test and lead vehicles performed the same evasive maneuver. Future research should investigate driver responses to CAD-initiated evasive maneuvers that are the same as the lead vehicle.

## Data availability statement

The raw data supporting the conclusions of this article will be made available by the authors, without undue reservation.

## Ethics statement

The studies involving human participants were reviewed and approved by Virginia Tech Institutional Review Board IRB #18-473. The patients/participants provided their written informed consent to participate in this study.

## Author contributions

MJ, JH, NB, and KK: study conception and design. NB: data collection. NB and MJ: analysis and interpretation of results and draft manuscript preparation. All authors contributed to the article and approved the submitted version.

## Funding

This project was funded by the Safety through Disruption (Safe-D) National University Transportation Center, a grant from the U.S. Department of Transportation – Office of the Assistant Secretary for Research and Technology, University Transportation Centers Program.

## Conflict of interest

The authors declare that the research was conducted in the absence of any commercial or financial relationships that could be construed as a potential conflict of interest.

## Publisher’s note

All claims expressed in this article are solely those of the authors and do not necessarily represent those of their affiliated organizations, or those of the publisher, the editors and the reviewers. Any product that may be evaluated in this article, or claim that may be made by its manufacturer, is not guaranteed or endorsed by the publisher.
